# A Study on the Nanostructural Evolution of Bi/C Anode Materials during Their First Charge/Discharge Processes

**DOI:** 10.3390/ma17051140

**Published:** 2024-02-29

**Authors:** Mengyuan Zhao, Weidong Cheng, Xin Wang, Huanyan Liu, Xiang Chen, Chaohui Wang, Yuan You, Zhaojun Wu, Bing Wang, Zhonghua Wu, Xueqing Xing

**Affiliations:** 1 College of Materials Science and Engineering, Qiqihar University, Qiqihar 161006, China; zmengyuan0320@163.com (M.Z.); xwang@ihep.ac.cn (X.W.); 18892258337@163.com (H.L.); 17754833098@163.com (X.C.); wch800209@126.com (C.W.); greatyouyuan@163.com (Y.Y.); 2 Institute of High Energy Physics, Chinese Academy of Sciences, Beijing 100049, China; wuzh@ihep.ac.cn; 3 Department of Practice Teaching and Equipment Management, Qiqihar University, Qiqihar 161006, China; wuzj@qqhru.edu.cn; 4 CAS Key Laboratory for Biomedical Effects of Nanomaterials and Nanosafety, Institute of High Energy Physics, Chinese Academy of Sciences, Beijing 100049, China; wangbing@ihep.ac.cn

**Keywords:** Bi/C composite, lithium-ion battery, SAXS, anode material

## Abstract

As a candidate anode material for Li-ion batteries, Bi-based materials have attracted extensive attention from researchers due to their high specific capacity, environmental friendliness, and simple synthesis methods. However, Bi-based anode materials are prone to causing large volume changes during charging and discharging processes, and the effect of these changes on lithium storage performance is still unclear. This work introduces that Bi/C nanocomposites are prepared by the Bi-based MOF precursor calcination method, and that the Bi/C nanocomposite maintains a high specific capacity (931.6 mAh g^−1^) with good multiplicative performance after 100 cycles at a current density of 100 mA g^−1^. The structural evolution of Bi/C anode material during the first cycle of charging and discharging is investigated using in situ synchrotron radiation SAXS. The SAXS results indicate that the multistage scatterers of Bi/C composite, used as an anode material during the first lithiation, can be classified into mesopores, interspaces, and Bi nanoparticles. The different nanostructure evolutions of three types of Bi nanoparticles were observed. It is believed that this result will help to further understand the complex reaction mechanism of Bi-based anode materials in Li-ion batteries.

## 1. Introduction

Lithium-ion batteries have gained popularity in recent years due to their long lifespan and high energy density [1,2,3]. They are widely used in electronic products such as smartphones, laptops, and electric vehicles [4,5]. Although significant progress has been made in developing lithium batteries with higher power, higher energy density, and longer life cycles, the demand for higher energy density and longer lifespan is increasing due to the introduction of electric vehicles and the development of high-performance electronic devices. Currently, graphite anode materials available on the market have a relatively low theoretical capacity. These materials are plagued by issues such as rapid capacity decay and poor safety performance, making it challenging to meet market demand during usage [6,7]. Therefore, anode materials play a crucial role in determining the performance and service life of lithium-ion batteries.

Researchers have shown great interest in Bi-metal and its compounds due to their high specific capacity, environmentally friendly nature, and simple synthesis methods [8]. Metallic Bi has a much higher volume-specific capacity (3800 mAh cm^−3^) compared to graphite anode (756 mAh cm^−3^). Its unique layered structure enables easy intercalation of lithium ions [9,10]. However, low conductivity and large volume expansion (~215%) hinder its practical applications in lithium-ion batteries during charging and discharging processes [11,12]. To solve this problem, researchers and scholars have prepared bismuth carbon composites with various structures, including core-shell structures [13,14], nanofibres [15,16], nanoplatelets [17], hierarchical structures [18], sandwich structures [12], egg-carton shapes [19], and porous structures [20,21]. These structures are employed to prevent Bi-agglomeration, reduce volume change, and promote microscopic reaction kinetics. The excellent performance of the Bi@C nanoplates electrode is attributed to the nanoscale Bi and amorphous carbon shell. Bi nanoparticles reduce the diffusion distance of lithium ions, while the amorphous carbon shell improves the conductivity of the electrode and suppresses the crushing and aggregation of Bi nanoparticles during the charging and discharging processes [17]. Among them, the egg-carton shape Bi/C anode material maintains a capacity of 523 mAh g^−1^ after 100 cycles at a current density of 100 mA g^−1^ [19]. The MOF-derived hierarchical micro/mesoporous nanostructured Bi@C anode material exhibits a high capacity of 556 mAh g^−1^ after 100 cycles at a current density of 100 mA g^−1^ [17].

In situ synchrotron radiation (SR) techniques, such as X-ray absorption spectroscopy [22], X-ray reflectivity [23], X-ray diffraction [24], and small angle X-ray scattering (SAXS) [25,26], have been used to study real-time structural changes in electrode materials of lithium-ion batteries. In situ electrochemical-SR combined techniques can be used to obtain information on structural changes, such as crystal transformation, changes in oxidation state, solid electrolyte interface (SEI) characterization, and lithium dendrite growth mechanisms during lithium-ion embedding/de-embedding [23,24,25]. For instance, the SR XRD technique investigates that the CuO/GO anode material displays a noticeable shift of the CuO(-511) Bragg peak to a higher angle due to Li^+^ doping in the CuO lattice during the first cycle. At the same time, SR SAXS can obtain qualitative information, such as particle shape, size and distribution, fractal dimension, and so on, from the fluctuation of electron density in nanoparticles. SAXS analysis reveals alterations in the formation and growth of SEI in CuO/GO electrode material as the lithiation dose increased, ultimately leading to cracking [26]. It has been demonstrated through operando grazing-incidence small-angle X-ray scattering (GISAXS) and X-ray diffraction (GIXD) that the evolution of the mesoporous structure of the metal oxide anode during battery operation is largely influenced by the initial mesoporous size [24]. The study of the nanostructural changes of the silicon phase in the silicon/graphite-based composite anode by SAXS shows that the additional lithium is stored in the silicon, and the silicon phase acts as a compensating reservoir for lithium capture. But, there is no adverse effect of large volume changes [27]. However, there are few reports on the nanostructural evolution of Bi/C electrodes during the lithiation/delithiation processes by the SR SAXS technique. In this work, Bi/C nanocomposites are prepared as the anode of lithium-ion batteries using Bi-MOF as a precursor, featured for a high capacity of 931.6 mA h g^−1^ at 100 mA g^−1^ after 100 cycles, and C as a buffer matrix for the volume expansion of Bi nanoparticles. The evolutions of the nanostructures of the Bi/C anode material at the electrode/electrolyte interface are analyzed using in situ electrochemical-SR SAXS techniques during their first charge/discharge processes.

## 2. Experimental Section

### 2.1. Materials

Bismuth nitrate pentahydrate [Bi (NO_3_)_3_∙5H_2_O, 99%], polyvinylidene fluoride (PVDF, ≥99.5%), 1,3,5-benzene tricarboxylic acid (H_3_BTC, 98%), Acetylene black (99.9%), methanol (99.9%) and 1-Methyl-2-pyrrolidinone (NMP, 99.9%) were purchased from Shanghai Maclin Biochemical Co., Ltd., Shanghai, China and used without further purification.

### 2.2. Preparation of Bi-Based MOFs

Bismuth-based MOFs were prepared using the solvothermal method. Firstly, 750 mg of H_3_BTC was dissolved in 60 mL of anhydrous methanol, and 150 mg of Bi (NO_3_)_3_∙5H_2_O was added to the above solution. After 30 min of stirring at room temperature, the mixed solution was transferred to a 100 mL Teflon-lined stainless-steel autoclave and reacted at 120 °C for 12 h. The product was a white precipitate, collected by centrifugation at 4000 r/min, washed with anhydrous methanol 3 times, and dried at 60 °C overnight in a vacuum drying oven [28].

### 2.3. Preparation of Bi/C Composite

**Figure 1:**
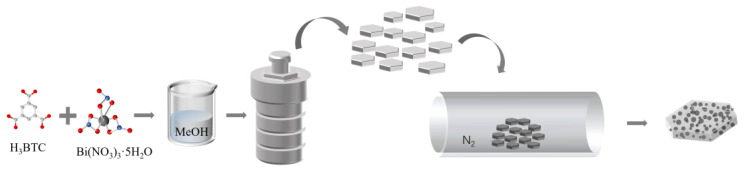
The schematic diagram of Bi/C synthesis.

The preparation process of Bi/C material is illustrated in Figure 1. The dried powder, Bi-based MOF, was calcined at 700 °C for a different time under an N_2_ atmosphere to enable the formation of a Bi/C composite. After carbonization, the black powder was cooled slowly under inert conditions.

### 2.4. Material Characterization

The morphology and microstructure of Bi/C materials were observed by transmission electron microscopy (TEM, H-7650, Hitachi Ltd., Tokyo, Japan) and scanning electron microscopy (SEM, S-3400, Hitachi Ltd., Japan). X-ray diffraction patterns (XRDs) were obtained by using a SmartLab-type X-ray diffractometer with a Cu Kα radiation source (λ = 0.15418 nm). Raman spectra were collected using a spectrophotometer (In Via, 113 Renishaw, Wotton-under-Edge, UK) equipped with a 532 nm laser. X-ray photoelectron spectroscopy (XPS) was measured on the ESCALAB 250Xi X-ray photoelectron spectrometer to determine the elemental valence state of the product. Thermogravimetric analysis (TG) was tested on a Diamiand thermal analyzer from room temperature to 800 °C, 10 °C/min in a nitrogen atmosphere. Nitrogen adsorption–desorption isotherms were obtained by using a specific surface area and pore size analyzer (BSD-PS1, Beishide Instrument Technology, Beijing, China).

### 2.5. Electrochemical Measurements

The preparation method of the working electrode was as follows: the active material, acetylene black, and PVDF were mixed in a mass ratio of 7:2:1, fully ground, and NMP was added to adjust the viscosity of the slurry. The carbon cloth was used as the current collector, and the slurry was evenly applied to the current collector. The electrode mass loading was about 3 mg/cm^2^. The lithium sheet was used as the counter electrode, and the electrolyte was a mixed solution of lithium hexafluorophosphate (LiPF_6_) dissolved in ethylene carbonate (EC), dimethyl carbonate (DMC), and methyl ethyl carbonate (EMC) with a volume ratio of 1:1:1. The diaphragm was Celgard 2400 microporous polypropylene membrane. The cyclic voltammetry (CV) test was conducted using a Chenhua Electrochemistry Workstation (CHI600C) in the voltage range of 0.01~3 V, while the AC impedance spectrum (EIS) was recorded within a frequency range of 10 MHz to 100 kHz. The electrochemical performances of the Bi/C material, including its cycle stability and rate capability, were evaluated using a LAND battery test system.

### 2.6. In Situ SAXS Experiment

The experiment was carried out at a BSRF 1W2A small angle scattering beamline station. The X-ray incident wavelength was 0.154 nm. Other experimental parameters include a storage ring operating voltage of 2.5 GeV, a current of about 200 mA, a detector model Mar165 (pixel range of 2048 × 2048, pixel size of 79 µm × 79 µm), and a distance of 1480 mm between detector and sample. SAXS experiment was conducted simultaneously with constant current charging and discharging tests. The voltage range is 0.4–3 V, and the SAXS exposure time is 5 s. The SAXS image is converted into a one-dimensional curve by the software Fit-2d (12.077_i686_WXP.exe).

## 3. Results and Discussion

**Figure 2:**
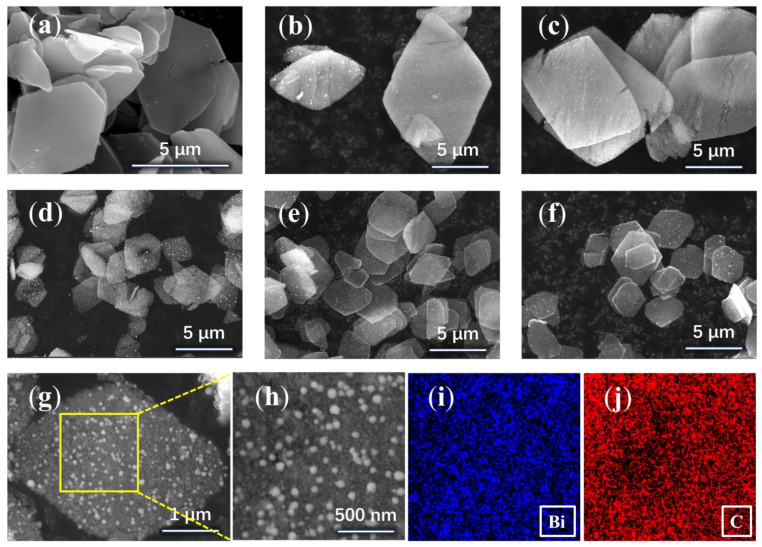
SEM images of the (**a**) Bi-MOF, (**b**) Bi/C-30, (**c**) Bi/C-60, (**d**) Bi/C-90, (**e**) Bi/C-120, (**f**) Bi/C-150, (**g**) Bi/C-90, (**h**–**j**) EDS mapping images of Bi/C composite.

A solvothermal method was used to prepare Bi-MOF with a smooth surface lamellar structure in methanol solvent using bismuth nitrate pentahydrate as a bismuth source and 1,3,5-benzene tricarboxylic acid as a ligand (Figure 2a). In the next step, the calcination method was used, and the prepared Bi-MOF samples were dried and then calcined under a nitrogen atmosphere for different durations. To prevent oxidization, they were kept in an inert state until they were cooled down to room temperature after the calcination was completed. Eventually, the formed Bi/C composites maintained the MOF lamellar structure with the appearance of granular spheres on the material surface as shown in Figure 2b–g of the SEM images.

Figure 2b–f show the Bi/C material calcined for 30 min, 60 min, 90 min, 120 min, and 150 min, which are denoted as Bi/C-30, Bi/C-60, Bi/C-90, Bi/C-120, and Bi/C-150, respectively. It can be seen from the figures that the Bi/C composites maintain the original lamellar carbon skeleton. After calcination, small spherical particles appear on the surface of Bi-MOF samples at 30 min. As the calcination time increases, cracks appear in the sample at 60 min and gradually decompose into thin slices of about 3–4 µm at 90 min. There is no significant change in the samples as the calcination time continued to increase. Figure 2g shows the SEM image of a Bi/C-90 nanosheet with a scale bar of 1 µm. Figure 2h–j show the region (yellow box) of the EDS spectra taken from Figure 2g for testing and the EDS mapping of Bi and C elements, respectively. It illustrates the uniform distribution of Bi and C elements in the nanosheet matrix, which is beneficial for improving structural stability and electronic conductivity.

**Figure 3:**
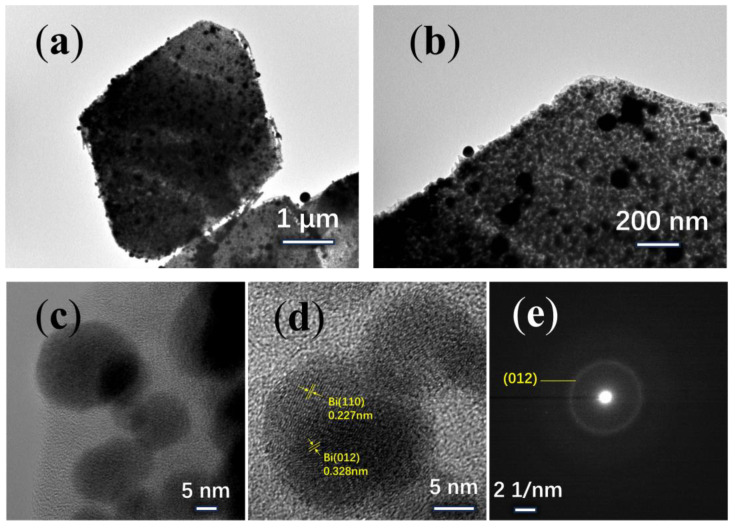
TEM images of the Bi/C composite: (**a**–**c**) Bi/C-90, (**d**) HRTEM, (**e**) SAED.

The TEM images with different scale bars show that the nanoparticles are uniformly embedded in the carbon skeleton (Figure 3a–c). The interfacial d-spacing of the nanoparticles was 0.328 nm, and 0.227 nm, respectively, which corresponded to the (012), and (110) crystal faces of Bi nanoparticle (Figure 3d). As shown in the electron diffraction pattern (Figure 3e), the diameter of the diffraction ring is chosen as 0.152 (1/nm), resulting in d = 0.328 nm, which corresponds to the (012) crystal face. It indicates that the Bi nanoparticle in the sample is a single crystal.

**Figure 4:**
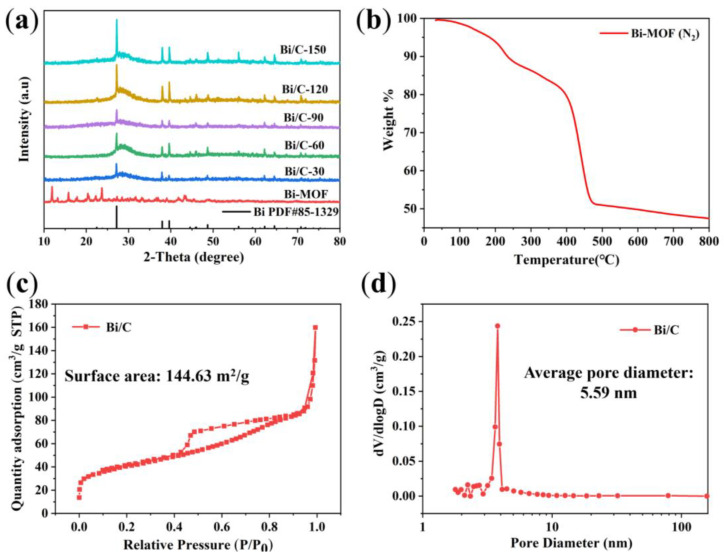
(**a**) XRD pattern and (**b**) TG curves of the Bi-MOF sample, (**c**) N_2_ adsorption and desorption isotherm and (**d**) pore size distribution of Bi/C-90 composite.

XRD is used to analyze the physical phase changes of Bi/C composites. It is evident that after annealing in nitrogen at 700 °C, the XRD patterns of the resulting samples show the formation of crystalline peaks compared to the initial Bi-MOF sample (Figure 4a).

The XRD analysis reveals the characteristic peaks at 27.2°, 37.9°, 39.6°, 48.7°, 62.2°, and 64.5°, which correspond to the (012), (104), (110), (202), (116), and (122) crystallographic planes of single-crystal Bi (PDF#85-1329), respectively. The halo peaks between 20 and 40° are associated with amorphous carbon. An increase in calcination time leads to higher crystallinity. As shown in Figure 4b, the TG shows the conversion of Bi-MOFs into Bi/C material in nitrogen. The TG curve shows three distinct mass loss steps. The first stage (room temperature to 200 °C) and the second stage (250–400 °C) can be attributed to the removal of physisorbed water and methanol solvent, respectively. The third stage (400–450 °C) results in a weight loss of approximately 30%, which is due to the decomposition and carbonation of the Bi-MOF material.

Porous structures can increase the contact area between electrolyte and electrode material, thereby improving the Li-ion migration [29]. For instance, microporous structures, such as metal foam structures, are added to the electrode material to enhance the contact area between the electrode and the electrolyte. The Bi/C-90 material’s porosity is determined using N_2_ adsorption and desorption isotherms (Figure 4c). The surface area of Bi/C-90 material is calculated to be 144.63 m^2^/g using the Brunauer–Emmett–Teller (BET) analysis. The N_2_ adsorption isotherm indicates that the mesopores of the Bi/C sample are well-developed. The pore size distribution, calculated using the Barrett–Joyner–Halenda (BJH) method, is shown in Figure 4d, confirming that the Bi/C-90 composite has a mesoporous structure with a uniform pore size of 5.59 nm. The data suggest that Bi/C possesses a sheet structure with uniformly embedded mesopores in the amorphous Bismuth-carbon skeleton, resulting in a large specific surface area.

**Figure 5:**
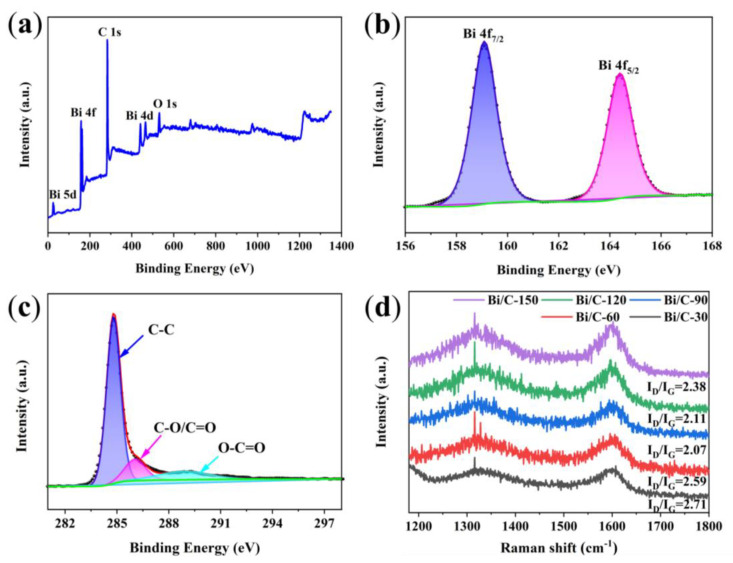
(**a**) XPS survey, (**b**) high-resolution Bi 4f spectra, (**c**) high-resolution C 1s spectra of the Bi/C-90, and (**d**) Raman spectra of the Bi/C composites.

XPS analysis is used to examine the chemical and surface electronic states of the Bi/C-90 composite. The XPS survey of Bi/C-90 (Figure 5a) reveals the characteristic peaks of Bi 4f, O 1s, and C 1s, indicating the presence of Bi, O, and C elements. And, the carbon exhibited a high degree of graphitization. The high-resolution Bi 4f spectrum shows a pair of characteristic peaks at binding energies 159.0 and 164.3 eV for Bi/C, corresponding to Bi 4f_7/2_ and Bi 4f_5/2_ for Bi^3+^ [30], respectively (Figure 5b). The high-resolution C 1s spectrum (Figure 5c) can be divided into three peaks at 284.8, 285.6, and 289.0 eV. The peak at 285.6 eV is related to C-O/C=O, and the other two peaks are attributed to the C-C and O-C=O bonds, respectively. To further investigate the degree of graphitization, Raman tests are performed. The Raman spectra of the Bi/C composites display a pair of characteristic peaks at 1350 and 1597 cm^−1^ (Figure 5d), corresponding to the D and G peaks of C atoms [31]. The D peak corresponds to carbon defects in the lattice and the G peak corresponds to the degree of carbonization of the material. The value of I_D_/I_G_ is obtained from the ratio of peak areas of D and G peaks. The I_D_/I_G_ values of Bi/C-30, Bi/C-60, Bi/C-90, Bi/C-120, and Bi/C-150 are 2.71, 2.59, 2.07, 2.11, and 2.38, respectively. Among the Bi/C composites, Bi/C-30 exhibits the highest I_D_/I_G_ value, indicating that the crystals of Bi/C-30 sample are more disordered and have more defects. With the increase in calcination time, defects are reduced, and the degree of graphitization and electrical conductivity of the sample are increased, which will be conducive to the embedding/de-embedding process of Li-ion and improve the rate capacity. The minimum I_D_/I_G_ value of Bi/C-90 should have better rate performance.

**Figure 6:**
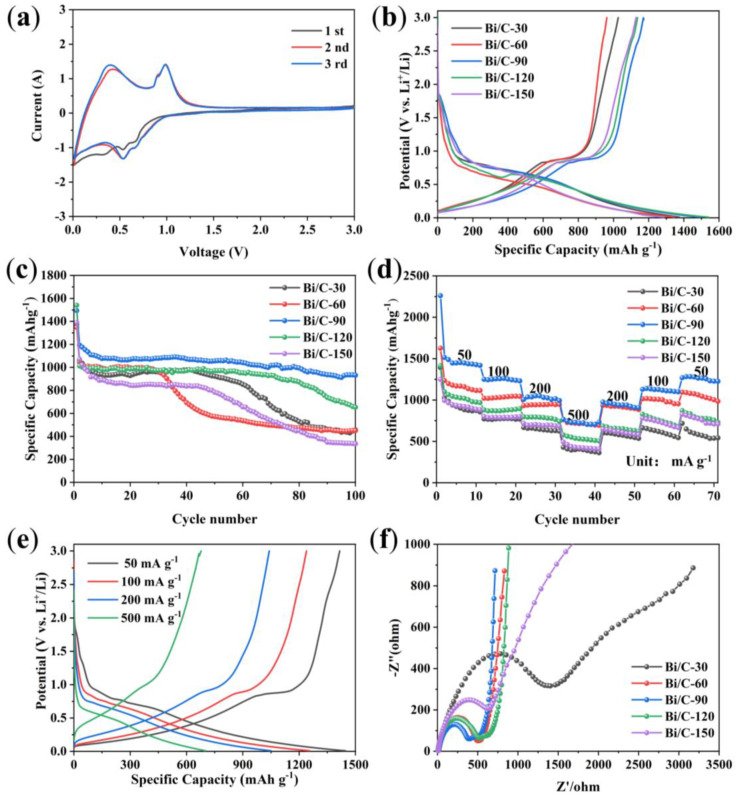
(**a**) CV curves of the Bi/C-90, (**b**) initial galvanostatic charge/discharge curves, (**c**) cycle performance at 100 mA g^−1^, (**d**) rate capability, (**e**) galvanostatic charge/discharge curves of the Bi/C-90 at various current densities, and (**f**) Nyquist plots.

CV curves from the first to third cycles are obtained using a scan rate of 0.1 mV s^−1^ in the range of 0.01~3.0 V (Li^+^/Li) as shown in Figure 6a. There are two redox pairs at 0.54 V and 0.65 V. These two peaks correspond to the lithiation process (cathodic process), which produces LiBi and Li_3_Bi from Bi-metal alloys [32,33,34]. During the reduction process, two additional peaks are observed at 0.90 V and 0.98 V, which correspond to the reduction in the alloy to Bi. The CV curves of the second and third cycles exhibit high reversibility with significant overlap in shape and intensity. Regarding the first cycle, the initial excess current compared to the following cycles is attributed to the formation of the SEI layer. Figure 6b shows the initial constant-current charge/discharge curves of Bi/C anode materials at a current density of 100 mA g^−1^. The initial discharge capacity of Bi/C-120 is 1540.5 mAh g^−1^, which is higher than that of Bi/C-30 (1374.6 mAh g^−1^), Bi/C-60 (1346.6 mAh g^−1^), Bi/C-90 (1497.1 mAh g^−1^), or Bi/C-150 (1285.7 mAh g^−1^). The corresponding initial Coulombic efficiencies are 74.6%, 71.4%, 78.2%, 73.8% and 87.7% for Bi/C-30, Bi/C-60, Bi/C-90, Bi/C-120 and Bi/C-150, respectively. Additionally, all Bi/C materials have clear plateaus near 0.9 V in the charge curve and near 0.5 and 0.6 V during discharge, which are consistent with the CV results.

The cycling stability of lithium-ion batteries is an important parameter for measuring battery performance. Poor cycling performance indicates severe lithium-ion consumption within the anode material, which will shorten the battery’s lifespan. The cycling performances of Bi/C anode materials at 100 mA g^−1^ are shown in Figure 6c. After 100 cycles, Bi/C-90 shows the best cycling stability with a capacity retention of 931.6 mAh g^−1^ and a capacity retention of 62.4%. In comparison, the capacity retention rates of Bi/C-30, Bi/C-60, Bi/C-120, and Bi/C-150 are 33.1%, 33.4%, 42.2%, and 24.1%, respectively. The excellent cycling performance of the Bi/C-90 anode material suggests that its preparation conditions are more conducive to mitigating the volumetric expansion of Bi nanoparticles during lithiation. Figure 6d illustrates the rate capabilities of Bi/C anode materials. The reversible capacities of Bi/C-90 at 100, 200, and 500 mA g^−1^ are 1239.2, 1040.6, and 700.5 mAh g^−1^, respectively (Figure 6e). When the current density is relaxed to 50 mA g^−1^, Bi/C-90 has a high capacity of 1415 mAh g^−1^, indicating excellent rate capability and reversibility. The electrochemical impedance spectra of Bi/C samples (Figure 6f) show that Bi/C-90 has the lowest charge transfer resistance, faster electrochemical reaction rate, and better diffusion capability. It can be seen that the Bi/C-90 composite has excellent electrochemical properties, which is conducive to the study of high-performance anode materials for Li-ion batteries.

**Figure 7:**
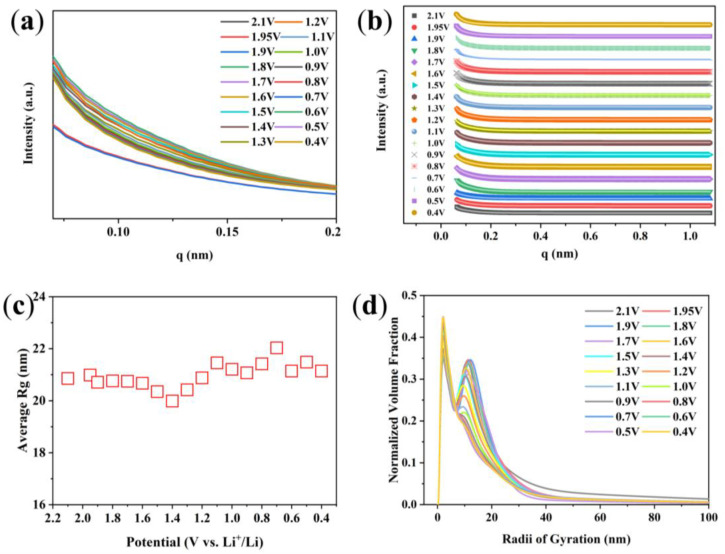
(**a**) The SAXS curves of Bi/C-90 anode material during the first discharge process, (**b**) SAXS experimental intensities (symbols) versus calculated values (solid lines), (**c**) average Rg variation of scattered nanoparticles, and (**d**) normalized volume fraction of scatterers during the initial discharge obtained by the TBT method.

The in situ SR SAXS experiment is used to study the structural evolution of Bi/C electrode material during the first cycle charge and discharge processes. One-dimensional SAXS curves are obtained by using the Fit-2d software processing. Figure 7a shows the SAXS curves measured during the first discharge of the Bi/C-90 anode material at a current density of 100 mA g^−1^. As can be seen from the figure, the variation in the scattering intensity is obvious. The SAXS technique can also determine the size and distribution of the scattering nanoparticles. The SAXS-fitted curves agree with the experimental curves fitted by the Tangent-by-Tangent (TBT) method (Figure 7b). The evolution of the average radius of gyration (Rg) of the Bi/C-90 anode material with potential during the first discharge can be obtained from the fits (Figure 7c). The average Rg increases to a maximum value at a voltage of 0.7 V and then gradually decreases. It reflects the size variation of average scattered nanoparticles in the Bi/C-90 electrode material. Figure 7d shows the normalized volume fractions (NVFs) obtained from the TBT method analysis, i.e., the Rg size distributions of the Bi/C-90 anode material during the first discharge process. The NVFs exhibit a clear bimodal distribution. The bimodal distribution has two independent Rgs at approximately 2 nm and 10 nm, respectively.

**Figure 8:**
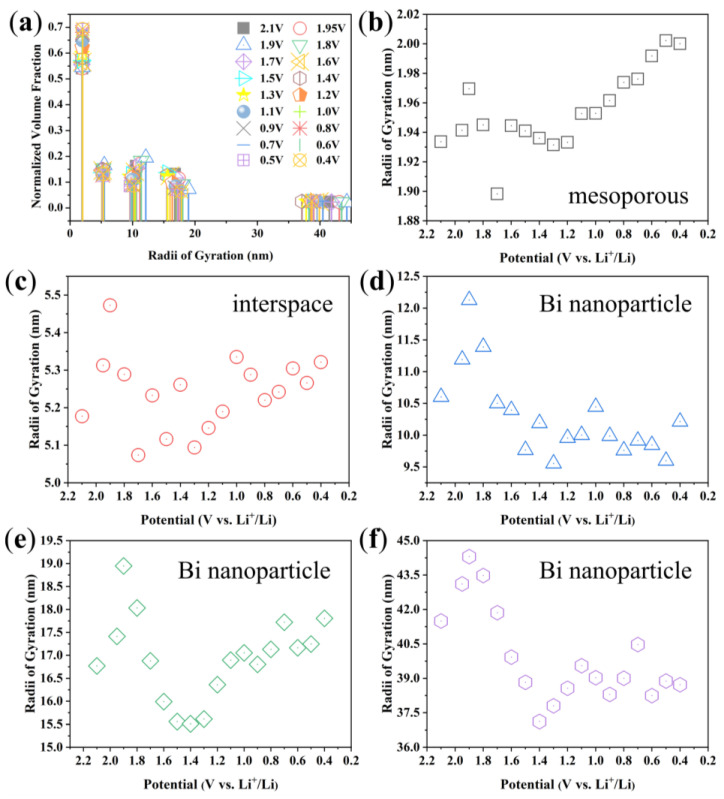
(**a**) The discrete distribution and (**b**–**f**) the physical Rgs of the polydisperse scatterers and their evolutions with the decreasing voltage.

It can be seen that the scatterers can be classified into five levels as shown in Figure 8a. The Rgs of the five scatterers and their evolutions with decreasing voltage are shown in Figure 8b–f, respectively. Obviously, the Rg evolutions of scatterers shown in Figure 8b and Figure 8d are consistent with the bimodal distribution of the NVF results, showing the importance of these two nanostructures during the charging and discharging processes. All levels of scatterers changed significantly in the first discharge process. Combined with the analysis of the nitrogen adsorption–desorption results, it can be seen that Figure 8b shows the mesoporous of Bi/C-90 composite, and the size of mesopores gradually increases during the discharge process. The Bi nanoparticles are stacked in the Bi/C matrix with interspaces. The size of the scatterer in Figure 8c coincides with the interspaces of the Bi nanoparticles. Figure 8d–f display three different sizes of Bi nanoparticles. There is a sharp increase in size from 2.1 V to 1.9 V, which is attributed to Li^+^ intercalation and the formation of SEI. The SEI increases in size with increasing lithiation dose, along with a volume increase of about 52%. Subsequently, the size starts to decrease at 1.9 V, and this may be due to the excessive increase in the volume of the nanoparticles, which resulted in cracks on their surface due to the tensile stress caused by the insertion of lithium. Therefore, it has an irreversible high capacity in the first discharge process. In Figure 8e,f, the scatterers display an increasing trend from 1.4 V to 0.4 V, which corresponds to the continued electrochemical reaction of exposed new Bi after the cracking of Bi nanoparticles, resulting in an increase in size. Figure 8d shows the smaller Bi nanoparticles do not change significantly after 1.4 V, which has higher dimensional stability. Assuming that the biggest Bi nanoparticles are spherical in Figure 8f, their diameters are approximately 100 nm.

**Figure 9:**
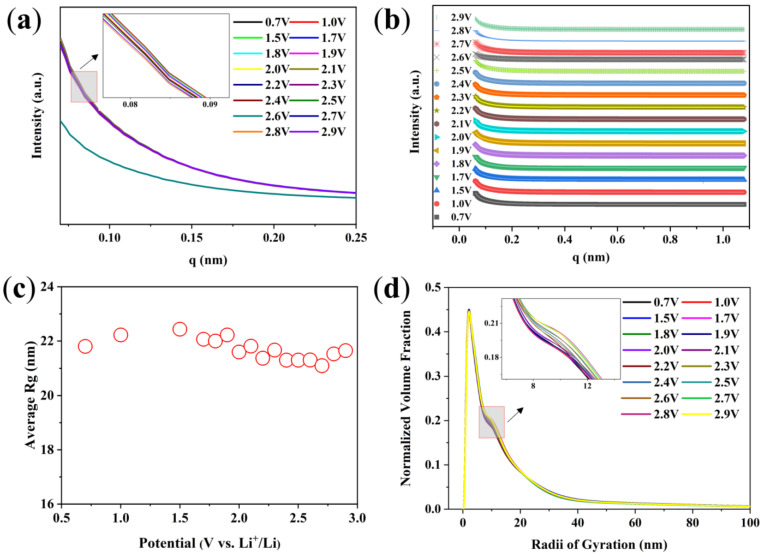
(**a**) SAXS curves of Bi/C-90 anode material during the first charge process, (**b**) SAXS experimental intensities (symbols) versus calculated values (solid lines), (**c**) average Rg variation of scattered nanoparticles, (**d**) NVFs of scatterers during the initial charge obtained by the TBT method.

**Figure 10:**
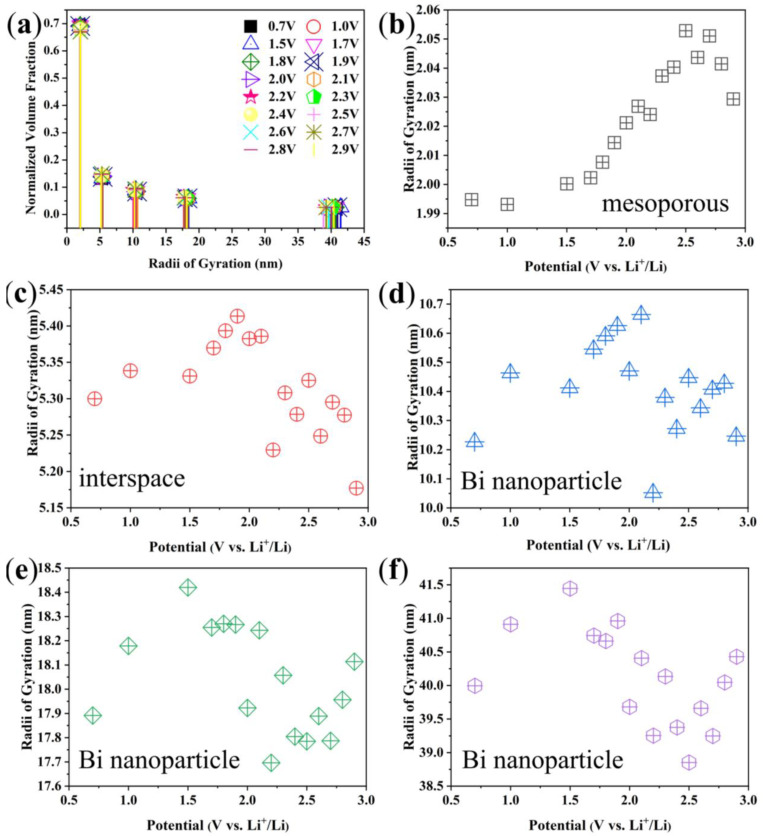
(**a**) Discrete distributions and (**b**–**f**) Rgs of these polydisperse scatterers during the first charging process and their variation with the increasing voltage.

Figure 9a shows the SAXS curves measured during the first charge of Bi/C-90 anode material at a current density of 100 mA g^−1^. The general trend is that the intensity decreases with increasing voltage (the inset of Figure 9a). The SAXS-fitted curves agree with the experimental curves fitted by the TBT method (Figure 9b). The evolution of the average Rg of the Bi/C anode material during the first charge process can be obtained from the fits (Figure 9c). The NVF undergoes a slight change (Figure 9d). The peak at about 2 nm decreases with increasing voltage and the peak at about 10 nm increases with the voltage (the inset of Figure 9d), which is opposite to the first discharge process. It can be seen that there are still five levels of scattering, as shown in Figure 10a. The mesopore of the Bi/C-90 composite increases with the increasing voltage (Figure 10b). The incomplete discharge of an open button battery leads to a rapid increase in voltage from 0.5 V to 1.5 V during the first charging process. Therefore, we speculate that due to this reason, the different scatterers exhibit an increasing trend within this voltage range as shown in Figure 10c–f. As Li^+^ deintercalates from 1.5 V to 3.0 V during the first charging process, the interspaces decrease (Figure 10c) and the different Bi nanoparticles (Figure 10d–f) all decrease with the increasing voltage.

In all the processes of this work, there are still some experimental factors that need to be carefully considered and further validated and resolved. Impurities, such as NMP, moisture, PVDF, and some metal ions, require special attention during the preparation process of anode material. They may reduce the electrochemical performance of the anode material. For example, the water in the active material will cause the insoluble separation reaction of PVDF during dispersion, which could affect the stability and dispersion effect of the slurry. Organic residual products of NMP can affect the normal film-forming reaction of electrolyte solvents. On the other hand, there is uncertainty in material preparation and testing. The thickness and uniformity of the slurry coating will greatly affect the capacity and cycling stability of the electrode material. When processing SAXS data, there is also a certain degree of error in the size obtained by our self-designed TBT software.

## 4. Conclusions

In conclusion, the Bi/C composites are prepared by the precursor calcination method using the Bi-MOF material as the precursor. The characterization results show that the lamellar structure of Bi/C composites helps to reduce the volume expansion of Bi nanoparticles during the charging and discharging processes and gives the Bi/C anode material better stability and cycling ability. The electrochemical results show that the Bi/C-90 anode has the best electrochemical performance in terms of cycling performance and multiplicity performance. In addition, the first charge/discharge processes are investigated using the SAXS technique. The SAXS technique shows that the Bi/C-90 electrode has undergone obvious nanostructural changes during the first charge/discharge process. During the initial discharging process, the two bigger Bi nanoparticles undergo three stages. As Li^+^ becomes embedded, the Bi nanoparticle size increases, leading to partial cracking. After the new Bi is exposed, Li^+^ is embedded again, causing the size to increase once more. In contrast, the smaller Bi particles undergo more steady change. Incomplete discharge of the open button battery may have a significant impact on the subsequent charging process. This work will help to further understand the complex reaction mechanism of nanostructured bismuth-based anode materials. The applications of in situ electrochemical-SR technologies to battery materials will be of great significance for the fundamental understanding of hierarchical structure and improved performance of electrode materials.

## Data Availability

Data are contained within the article.

## Nomenclature

SR	synchrotron radiation
SAXS	small angle X-ray scattering
SEI	solid electrolyte interface
GISAXS	grazing-incidence small-angle X-ray scattering
GIXD	grazing-incidence X-ray diffraction
TEM	transmission electron microscopy
SEM	scanning electron microscopy
XRD	X-ray diffraction patterns
XPS	X-ray photoelectron spectroscopy
TG	thermogravimetric analysis
CV	cyclic voltammetry
EIS	AC impedance spectrum
BET	Brunauer–Emmett–Teller
BJH	Barrett–Joyner–Halenda
Bi/C-30; Bi/C-60; Bi/C-90; Bi/C-120; Bi/C-150	material calcined for 30 min, 60 min, 90 min, 120 min, and 150 min
TBT	Tangent-by-Tangent
Rg	radius of gyration
NVFs	normalized volume fractions
